# Phosphoflow cytometry to assess cytokine signaling pathways in peripheral immune cells: potential for inferring immune cell function and treatment response in patients with solid tumors

**DOI:** 10.1186/s13046-023-02802-1

**Published:** 2023-09-23

**Authors:** Nicole J. Toney, Jeffrey Schlom, Renee N. Donahue

**Affiliations:** Center for Immuno-Oncology, Center for Cancer Research, National Cancer Institute, National Institutes of Health, Bethesda, MD USA

**Keywords:** Phosphoflow cytometry, Signaling, PBMC, Clinical response, Solid tumors

## Abstract

Tumor biopsy is often not available or difficult to obtain in patients with solid tumors. Investigation of the peripheral immune system allows for in-depth and dynamic profiling of patient immune response prior to and over the course of treatment and disease. Phosphoflow cytometry is a flow cytometry‒based method to detect levels of phosphorylated proteins in single cells. This method can be applied to peripheral immune cells to determine responsiveness of signaling pathways in specific immune subsets to cytokine stimulation, improving on simply defining numbers of populations of cells based on cell surface markers. Here, we review studies using phosphoflow cytometry to (a) investigate signaling pathways in cancer patients’ peripheral immune cells compared with healthy donors, (b) compare immune cell function in peripheral immune cells with the tumor microenvironment, (c) determine the effects of agents on the immune system, and (d) predict cancer patient response to treatment and outcome. In addition, we explore the use and potential of phosphoflow cytometry in preclinical cancer models. We believe this review is the first to provide a comprehensive summary of how phosphoflow cytometry can be applied in the field of cancer immunology, and demonstrates that this approach holds promise in exploring the mechanisms of response or resistance to immunotherapy both prior to and during the course of treatment. Additionally, it can help identify potential therapeutic avenues that can restore normal immune cell function and improve cancer patient outcome.

## Background

Tumor tissue biopsy allows for in-depth profiling of the primary tumor immune microenvironment at the time of surgical resection. However, tissue biopsies of metastatic lesions of patients with most solid tumors are often not available or difficult to obtain, and typically provide information only from a single lesion and time point in the evolution of a tumor mass. Utilizing “blood biopsies” to study the peripheral immune response in cancer patients is less invasive and more dynamic, allowing for monitoring over the course of treatment and disease, and can complement analysis of the tumor microenvironment.

Currently, investigation of the peripheral immune response is largely focused on quantification of peripheral immune cell frequencies and expression of cell surface markers using flow or mass cytometry, whole transcriptome sequencing, epigenetic changes, T and B cell receptor sequencing, and levels of serum and plasma factors [1]. Despite the ability to quantify in-depth profiles of peripheral immune cell subsets, focusing on frequencies and cell surface markers of immune cells alone may not provide sufficient insight into their function.

Cytokines can regulate the immune response through activation of key signaling pathways in immune cells [2]. The ability of immune cells to respond to cytokines therefore influences their function, and signaling in immune cells may reflect their ability to mount an anti-cancer response. Exploration of cytokine signaling in peripheral immune cells may provide insight into immune cell dynamics and altered immune cell function, and may help to predict cancer patient response to therapy.

Common methods to visualize activation of cellular signaling pathways include immunohistochemistry (IHC) and immunofluorescence (IF), western blot, immunoprecipitation (IP), real-time quantitative PCR (RT-qPCR), RNA sequencing, and flow cytometry. Certain staining techniques like IHC and IF are often not practical for the study of peripheral immune cells. Other methods, such as western blot, IP, RT-qPCR, and bulk RNA sequencing, can be used to study signaling within peripheral immune cells, but they do not allow for analysis of signaling in single cells. Single-cell RNA sequencing can be employed to examine signaling pathways in peripheral immune cells of cancer patients. One such study found that the interferon gamma (IFN-γ) signaling pathway was upregulated in CD4^+^ and CD8^+^ T cells of gastrointestinal cancer patients who responded to anti-PD-1 treatment, compared to non-responding patients [3]. However, this method can be costly and time-consuming, and does not easily allow for quantification of signaling response to cytokines.

**Fig. 1:**
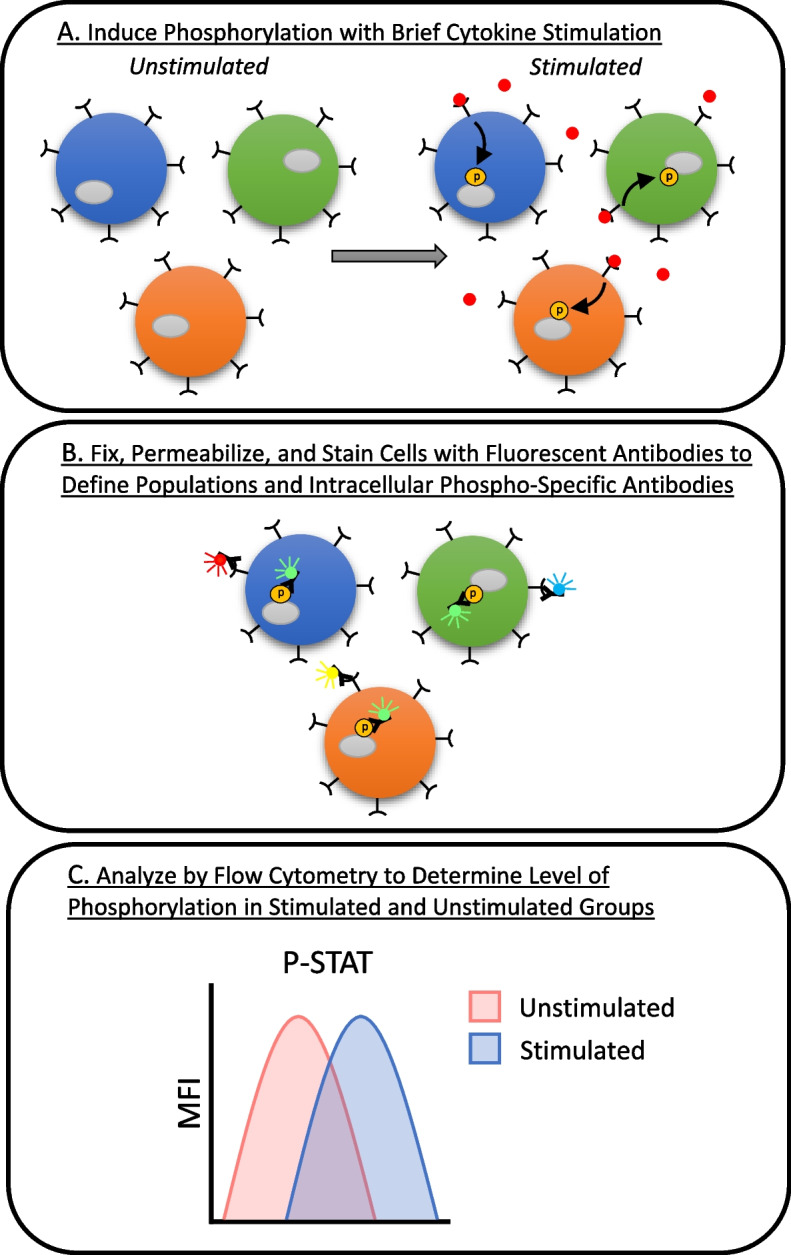
Phosphoflow cytometry method. A heterogeneous population of cells is briefly stimulated with cytokines, which bind to receptors and phosphorylate intracellular signaling proteins **A**. Cells are then fixed to preserve their phosphorylation status and stained with fluorescently labeled extracellular antibodies to define cell populations. Next, cells are permeabilized to allow access to intracellular proteins and stained with fluorescently labeled intracellular antibodies that specifically recognize phosphorylated forms of proteins of interest **B**. Labeled cells are then detected by flow cytometric analysis to determine phosphorylation levels in specific immune cell subsets of cytokine-stimulated and unstimulated groups **C**. MFI, mean fluorescent intensity. P-STAT, phosphorylated signal transducer and activator of transcription

Phosphoflow cytometry is a flow cytometry‒based method to analyze basal activation and sensitivity of immune cell signaling pathways to cytokine stimulation (Fig. 1). The protocol involves short-term stimulation of a diverse population of cells with cytokines. These cytokines attach to cell surface receptors causing phosphorylation of intracellular signaling proteins (Fig. 1A). Following this, the cells are fixed to maintain their phosphorylation state and then stained with extracellular antibodies labeled with fluorophores to define immune subset populations. Subsequently, the cells are permeabilized to access intracellular proteins and stained with fluorescently labeled intracellular antibodies, which specifically identify phosphorylated forms of proteins of interest (Fig. 1B). Through flow-cytometry analysis, the labeled cells are identified to determine phosphorylation levels in specific subsets of immune cells in both cytokine stimulated and unstimulated groups (Fig. 1C). This method allows for detection of phosphorylated signaling proteins within single cells [4–6].

Phosphoflow cytometry was first reviewed in 2004 by Krutzik et al. who discussed several studies using this technique, as well as the methods, technical considerations, and clinical applications existent at that time [7]. Krutzik and Nolan then went on to publish a comparison of staining techniques to optimize phosphoflow cytometry [4]. A review of phosphoflow cytometry was published in 2010 by Wu et al. [8], which centered on phosphoflow cytometry methods and preclinical applications for monitoring immune cells and cancer cells. The review also covered a limited number of studies that had employed phosphoflow cytometry to evaluate human immune responses, and highlighted several technical limitations existing at that time that needed to be addressed to improve the utility of phosphoflow cytometry as a potential immunomonitoring tool. At that time, these limitations included the need for (a) better fixation and permeabilization methods/reagents coupled with brighter fluorochrome conjugates to detect phosphorylated proteins, (b) flow cytometry technology compatible with small sample sizes, slow flow rates, and increased sensitivity, (c) identification of a wider panel of monoclonal antibody clones to identify various immune subsets that are compatible with the fixatives and permeabilization buffers needed for phosphoflow applications, (d) optimization and standardization of staining methods, and (e) new strategies to interpret and organize the increasing amount and complexity of experimental data on multiple signaling pathways in a single experiment. Since 2010, many of these limitations have been addressed, and subsequent studies, covered in the current review, have extended the utilization of phosphoflow cytometry in cancer immunology.

Phosphoflow cytometry has been used to investigate immune cell signaling and function in various peripheral blood mononuclear cell (PBMC) types such as regulatory T cells (Tregs) [9], total T cells [10], and B cells [11]. The method has been applied to various physiologic states, including aging [12], immunodeficiency [13], autoimmune disease [14, 15], and cancer, with the first application of this method to evaluate peripheral immune cells of patients with solid tumors published in 2004 by Lesinski et al. [16]. Methods for studying immune cells in cancer patient blood typically involve phenotyping by flow or mass cytometry and functional assays. Phenotyping provides information on the types and numbers of cells present and gives clues to their activation state, whereas functional assays inform on the ability of immune cells to perform their key functions. Functional assays of immune cells, however, often require large quantities of peripheral blood and can be time-consuming, and therefore may not always be feasible to perform in large numbers of patient samples. Phosphoflow cytometry informs on the ability of cells to respond to external cytokines by measuring activated signaling pathways; this signaling capacity may mirror function, and can be thought of as a bridge between phenotypic and functional assays.

Here, we present a current and comprehensive review of the application of phosphoflow cytometry in the field of cancer immunology. This review focuses on peripheral immune cells to identify activation and sensitivity of signaling pathways, evaluate the effect of various agents on the immune system, and potentially predict treatment response and patient outcome. Furthermore, we explore the use of phosphoflow cytometry in preclinical murine cancer models to demonstrate the potential applications of this method.

### Altered cytokine signaling in peripheral immune cells of cancer patients

Table 1 summarizes studies demonstrating the variances in cytokine signaling pathways in healthy donors and cancer patients, along with the immune cell types investigated. Below, we review studies reporting on these differences in cytokine signaling between healthy donors and cancer patients, and in cancer patients with varying plasma cytokine levels.

**Table 1:** Differences in basal- or cytokine-stimulated phospho-protein levels in cancer patients’ peripheral immune cells compared to healthy donor﻿s

Cancer type	Signaling pathway	Cell type	Increased or decreased in cancer patients vs healthy donors	Reference
Melanoma	IFNα – p-STAT1(Y701)	Lymphocytes	Decreased	[17]
Breast	IFNα – p-STAT1(Y701)	T cells, B cells, NK cells	Decreased	[18]
	IFNγ – p-STAT1(Y701)	B cells	Decreased	
Gastrointestinal	IFNα – p-STAT1(Y701)	T cells, B cells	Decreased	
	IFNγ – p-STAT1(Y701)	B cells	Decreased	
Melanoma	IFNα – p-STAT1(Y701)	T cells, B cells	Decreased	
	IFNγ – p-STAT1(Y701)	B cells	Decreased	
Breast	IL-6 p-STAT1(Y701)	CD4^+^ naïve T cells	Decreased	[19]
	IL-6 p-STAT3(Y705)	CD4^+^ naïve T cells	Decreased	
Hepatocellular	p-STAT3(Y705)	CD4^+^ and CD8^+^ T cells	Increased	[20]
Breast	IL-7 p-STAT5(Y694)	CD4^+^ and CD8^+^ T cells	Decreased	[21]

#### Altered interferon signaling in cancer patient peripheral immune cells

Antigen recognition, costimulation, and cytokine support are required to develop an effective T cell response [22]. IFN-γ is a cytokine primarily produced by activated natural killer (NK) and T cells and is essential for T cell function, including full activation, clonal expansion, and memory development. IFN-γ promotes T cell function through upregulation of MHC molecules [23, 24] and MHC class I and II processing machinery [25–27], promotes T cell differentiation [28, 29] and CD8^+^ T cell memory development [30], skews helper T cell responses toward a T helper 1 (Th1) phenotype [31], and improves motility and cytotoxicity of lymphocytes [31]. Interferon alpha (IFN-α) is a type I IFN that plays a key role in the innate immune response to viral infection and enhances cross-priming of CD8^+^ T cells exposed to antigen [32, 33]. IFNs bind to specific receptors on the surface of cells and activate signaling pathways that lead to the transcription of interferon-stimulated genes (ISGs). The signaling pathways activated by IFN-α and IFN-γ are complex and involve multiple signaling proteins, including Janus kinases (JAKs) and signal transducers and activators of transcription (STATs), including STAT1 [34].

Reduced IFN-α and IFN-γ signaling have been observed in peripheral blood lymphocytes from cancer patients. Downregulation in ISGs was found in T and B cells from melanoma patients compared to 12 healthy controls using single cell gene expression profiling [17]. Using phosphoflow cytometry to determine signaling response to IFN-γ, IFN-α, and IFN-β, phosphorylated STAT1 (p-STAT1) was reduced in lymphocytes stimulated only by IFN-α in melanoma patients compared to healthy controls, with these differences observed in CD8^+^ and CD4^+^ T cells, but not B cells. Upon IFN-α stimulation, ISGs remained lower in melanoma patients compared to healthy controls. The mechanism for the reduced IFN signaling was investigated by measuring gene expression levels of components of the IFN signaling pathway including STAT2, JAK1, JAK2, and Tyk2, which were not significantly different between healthy donor and melanoma patient lymphocytes. Therefore, the mechanism for impaired IFN signaling in T cells from melanoma patients was not determined.

IFN signaling has also been assessed in peripheral blood lymphocytes (including total, naïve and effector memory T cells, B cells, and NK cells) from patients with breast cancer, melanoma, and gastrointestinal cancer via RT-qPCR, phosphoflow cytometry, and functional assays [18]. ISGs, including STAT1, were found to be downregulated in blood lymphocytes from breast cancer patients compared to healthy controls via RT-qPCR. Reduced IFN-α and IFN-γ stimulation of p-STAT1 was identified by phosphoflow cytometry in cancer patient peripheral blood lymphocytes compared to healthy controls. IFN-α-induced p-STAT1 was impaired in B, T and NK cells from all cancer groups. IFN-γ-induced p-STAT1 was diminished in B cells, but not T and NK cells, from all cancer groups. Defective signaling was present in memory, effector, and naïve T cells from melanoma patients. There was no difference in impaired IFN-α and IFN-γ phosphorylation of STAT1 between early- and late-stage breast cancer patients, or between chemotherapy-treated and untreated breast cancer patient groups. Finally, the authors showed that the ability of T cells to be activated by anti-CD3/CD28 stimulation was reduced in breast cancer patients compared to healthy donors. NOS1 secretion by melanoma cells is one mechanism that has been identified as contributing to dysregulated IFN signaling through in vitro analysis [35]. The effects of soluble factors released by 12 melanoma cell lines on IFN signaling in healthy donor PBMCs were investigated using a Transwell system. Following 1 week of co-culture, PBMCs were stimulated with IFN-α and p-STAT1 induction was quantified by phosphoflow cytometry. NOS1 was identified as an inhibitory factor released by melanoma cells, which led to dysfunctional IFN-α p-STAT1 signaling in PBMCs.

Altogether, these studies using phosphoflow cytometry reveal that cancer patients, including those with melanoma and breast cancer, exhibit defects in IFN-α and IFN-γ signaling in their peripheral blood lymphocytes, which is evidenced by reduced activation of p-STAT1. This impairment is observed in various immune cell types, and the exact mechanism of T cell dysfunction in patients remains unknown.

#### Altered interleukin 6 signaling in cancer patients’ peripheral immune cells

Interleukin 6 (IL-6) is a cytokine that plays a central role in the immune system, influencing the activation and differentiation of immune cells and stimulation of acute phase responses [36]. IL-6 signaling is initiated when IL-6 binds to its receptor (IL-6R) on the surface of cells, leading to the phosphorylation and activation of JAK1 and STAT3. Activated STAT3 translocates to the nucleus and regulates the transcription of target genes. IL-6 signaling is necessary for proper T cell function; it is a costimulatory factor for T cells and promotes proliferation [37] and T cell survival [38], induces the initial production of IL-4 in CD4^+^ T cells, polarizing them to an effector T helper 2 (Th2) phenotype [39], aids effector T cells in overcoming Treg-mediated suppression, supports CD4^+^ T cell memory development [40], and skews T cell differentiation towards a Th17 phenotype and away from a Treg phenotype [41]. However, IL-6 is also a pleiotropic proinflammatory cytokine that reflects a negative prognosis in cancer patients, and prolonged signaling can lead to T cell dysfunction, thereby suppressing anti-tumor immune responses [42].

Impaired IL-6 signaling has been detected in peripheral T cells from both non-small cell lung cancer (NSCLC) patients with high vs low plasma IL-6 concentrations, and in breast cancer patients compared to healthy donors. In patients with NSCLC, the investigators sought to determine differences in T cells between patients with high levels of plasma IL-6 (> median of 6.41 pg/ml) compared to low levels (< 6.41 pg/ml), as high levels of IL-6 are associated with poor prognosis in these patients [43]. They found that patients with high levels of IL-6 had a higher percentage of peripheral Tregs and higher expression of PD-1 on both CD4^+^ and CD8^+^ T cells. The responsiveness of peripheral T cells to IL-6 was assessed from patients with high and low levels of plasma IL-6 using phosphoflow cytometry by measuring p-STAT1, p-STAT3, and p-ERK1/2. Patients with high IL-6 levels had lower activation of p-STAT1 in CD4^+^ T cells. No differences in induction of p-STAT3 or p-ERK1/2 was noted between the two groups. Furthermore, in vitro experiments were conducted using PBMCs from a healthy donor, where constant exposure to high levels of IL-6 was found to attenuate STAT signaling in T cells, leading to weaker signaling responses through STAT1, STAT3, and MAPK1/2 in CD4^+^ T cells.

**Fig. 2:**
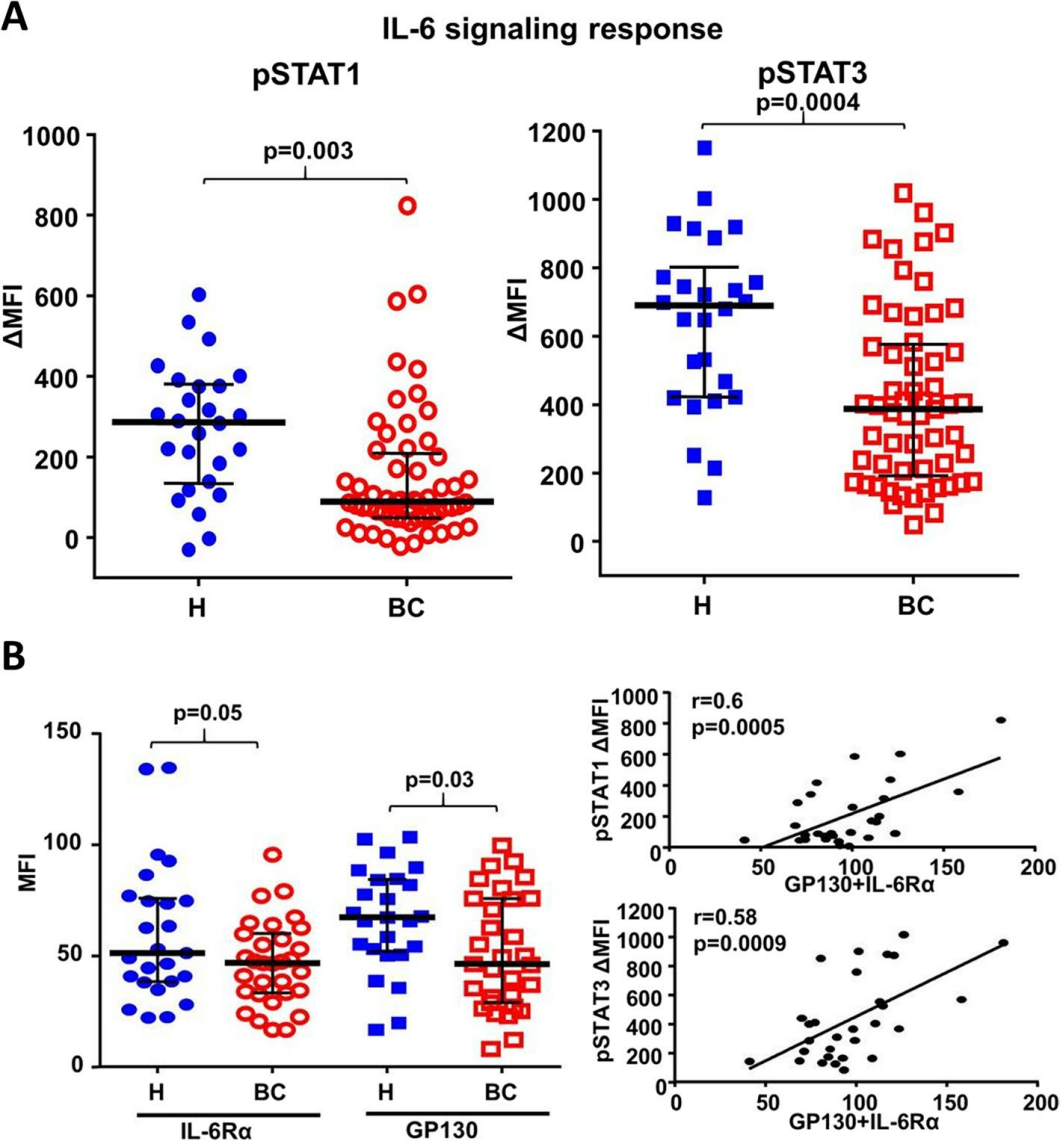
IL-6 signaling responsiveness in breast cancer patients’ CD4^+^ naïve T cells compared to healthy donors measured by phosphoflow cytometry. Median fluorescent intensity (MFI) of p-STAT1 and p-STAT3 between CD4^+^ naïve T cells stimulated with IL-6 minus unstimulated in healthy donors and breast cancer patients **A**. Flow-cytometry expression of IL-6Rα and GP130 on CD4^+^ naïve T cells in healthy donors and breast cancer patients, and the correlation with IL-6 signaling responsiveness of p-STAT1 and p-STAT3 **B**. Modified from Wang et al., Cancer Res. 2017 [19]. Copyright© 2017. American Association for Cancer Research

The responsiveness of IL-6 signaling in peripheral blood T cells has also been evaluated in breast cancer patients compared to healthy donors [19]. Treatment-naïve breast cancer patients and age-matched healthy donors were assessed for responsiveness of peripheral T, B, NK, and myeloid cells to IL-6 using phosphoflow cytometry. IL-6-induced p-STAT1 and p-STAT3 were lower in naïve CD4^+^ T cells from breast cancer patients compared to healthy donors (Fig. 2A). Peripheral levels of IL-6 were not elevated in this cohort of breast cancer patients compared to healthy donors, and no correlation was found between impaired IL-6 signaling response in naïve CD4^+^ T cells and plasma IL-6 levels. However, IL-6 receptors IL-6Rα and gp130 were found to be reduced on naïve CD4^+^ T cells from breast cancer patients compared to healthy donors by flow cytometry, and expression levels correlated with signaling responsiveness to IL-6, identifying a potential mechanism of impaired signaling (Fig. 2B). Furthermore, elevated mRNA level of ADAM19, which can cleave IL6-Rα, was found in breast cancer patient T cells.

Aberrant p-STAT3 expression has been identified in peripheral CD4^+^ and CD8^+^ T cells and related to development of hepatocellular carcinoma (HCC) [20]. In this study, basal levels of p-STAT3, measured by phosphoflow cytometry, were higher in peripheral blood CD4^+^ and CD8^+^ T cells of HCC patients compared to healthy controls. Serum cytokine levels of IL-4, IL-6, and IL-10 were increased, and IFN-γ levels were decreased in HCC patients compared to healthy controls. IFN-γ levels and IFN-γ/IL-4 ratio negatively correlated with p-STAT3 expression in CD4^+^ and CD8^+^ T cells in HCC patients, whereas levels of IL-4, IL-6, and IL-10 positively correlated with p-STAT3 levels.

Overall, these studies highlight the importance of balanced IL-6 signaling in T cell function and demonstrate the significance of impaired IL-6 signaling in various cancer types, including breast cancer, NSCLC, and HCC.

#### Altered interleukin 7 signaling in cancer patients’ peripheral immune cells

Interleukin 7 (IL-7) plays a role in the development, maintenance, and function of immune cells, particularly T and B cells, and is required for T cell development and memory function [44]. IL-7 signaling is activated when IL-7 binds to its receptor (IL-7R) on the surface of cells, leading to the phosphorylation and activation of JAK1 and STAT5. Activated STAT5 translocates to the nucleus and regulates the transcription of target genes.

Impaired IL-7 signaling was found in T cells from breast cancer patients [21]. Using phosphoflow cytometry, cancer patients’ PBMCs had lower constitutive p-STAT5 levels in CD4^+^ and CD8^+^ T cells compared to healthy donors. The signaling responsiveness of p-STAT5 to IL-7 stimulation was tested in CD4^+^ and CD8^+^ T cells; while all healthy donors (19/19) responded to IL-7, only 6/19 breast cancer patients responded to cytokine stimulation. In addition, IL-7Rα expression was reduced on peripheral CD4^+^ T cells from breast cancer patients compared to healthy donors. A functional assay testing immune effector function was carried out to corroborate and extend the phosphoflow cytometry findings. Intracellular cytokine production of IFN-γ and IL-2 upon PMA/lonomycin or T cell receptor (TCR) cross-linking stimulation was reduced in CD4^+^ and CD8^+^ T cells of breast cancer patients (9/19) compared to healthy donors’ (19/19) PBMCs, further indicating impaired function of these cells.

Overall, altered IFN, including both IFN-α and IFN-γ responses, IL-6, and IL-7 signaling pathways have been noted in cancer patients’ peripheral immune cells compared to those of healthy donors. Further investigation is needed into the prevalence, cause, and consequences of this varied cytokine signaling in cancer patients’ PBMCs.

### Comparisons between peripheral blood immune cell signaling and the tumor immune microenvironment

#### Differences between tumor-infiltrating lymphocytes and PBMCs

The relationship between peripheral and intratumoral immune cells is complex and not fully understood. Limited numbers of studies have assessed the correlation between signaling responsiveness in peripheral immune cells with that of intratumoral immune cells. The few studies that have attempted this comparison have found both similarities and differences in signaling response in the two domains. In one study, suppressed cytokine signaling was found in intratumoral lymphoma T cells compared to peripheral T cells [45]. Using phosphoflow cytometry, reduced IL-4, IL-10, and IL-21-induced p-STAT6 and p-STAT3 were identified in tumor infiltrating lymphocytes (TILs) in follicular lymphoma tumors. In particular, CD4^+^CD45RO^+^CD62^−^ follicular lymphoma TILs were unresponsive to cytokines; this was not observed in the autologous peripheral blood subset. In addition, CD4^+^PD1^hi^ follicular lymphoma TILs lost their cytokine responsiveness, whereas PD-1^neg^ TILs had normal cytokine signaling.

Signaling differences have also been noted in peripheral and intratumoral T cells from colorectal cancer (CRC) patients [46]. Peripheral blood and paired tumor tissue from 63 CRC patients and peripheral blood from 33 healthy donors were evaluated by phosphoflow cytometry analysis for IL-6, IL-10, and IL-2-induced phosphorylation of p-STAT1, p-STAT3, and p-STAT5, respectively, in helper T, Treg, and cytotoxic T cell subsets. The signaling response to cytokines in TILs tended to be lower compared to CRC patient and healthy donor PBMCs. IL-2-induced p-STAT5 in Tregs was the only signaling pathway with no difference between healthy donor PBMC and cancer patient PBMC and TIL groups. Certain signaling pathways were increased in cancer patient PBMCs (IL-10-induced p-STAT3 in helper T, Treg, and cytotoxic T cells) while others were decreased (IL-6 induced p-STAT1 in helper T, Treg, and cytotoxic T cells) compared to healthy control PBMCs.

Overall, these studies in both lymphoma and colorectal cancer patients have demonstrated differences in signaling responsiveness between peripheral and intratumoral immune cells, suggesting altered cytokine signaling pathways in TILs compared to their peripheral counterparts. It should be noted that in these and other studies, the differences or similarities observed could be due to the time of sampling of both tumor tissue of primary or metastatic lesions and PBMCs.

#### Similarities between TILs and PBMCs

**Fig. 3:**
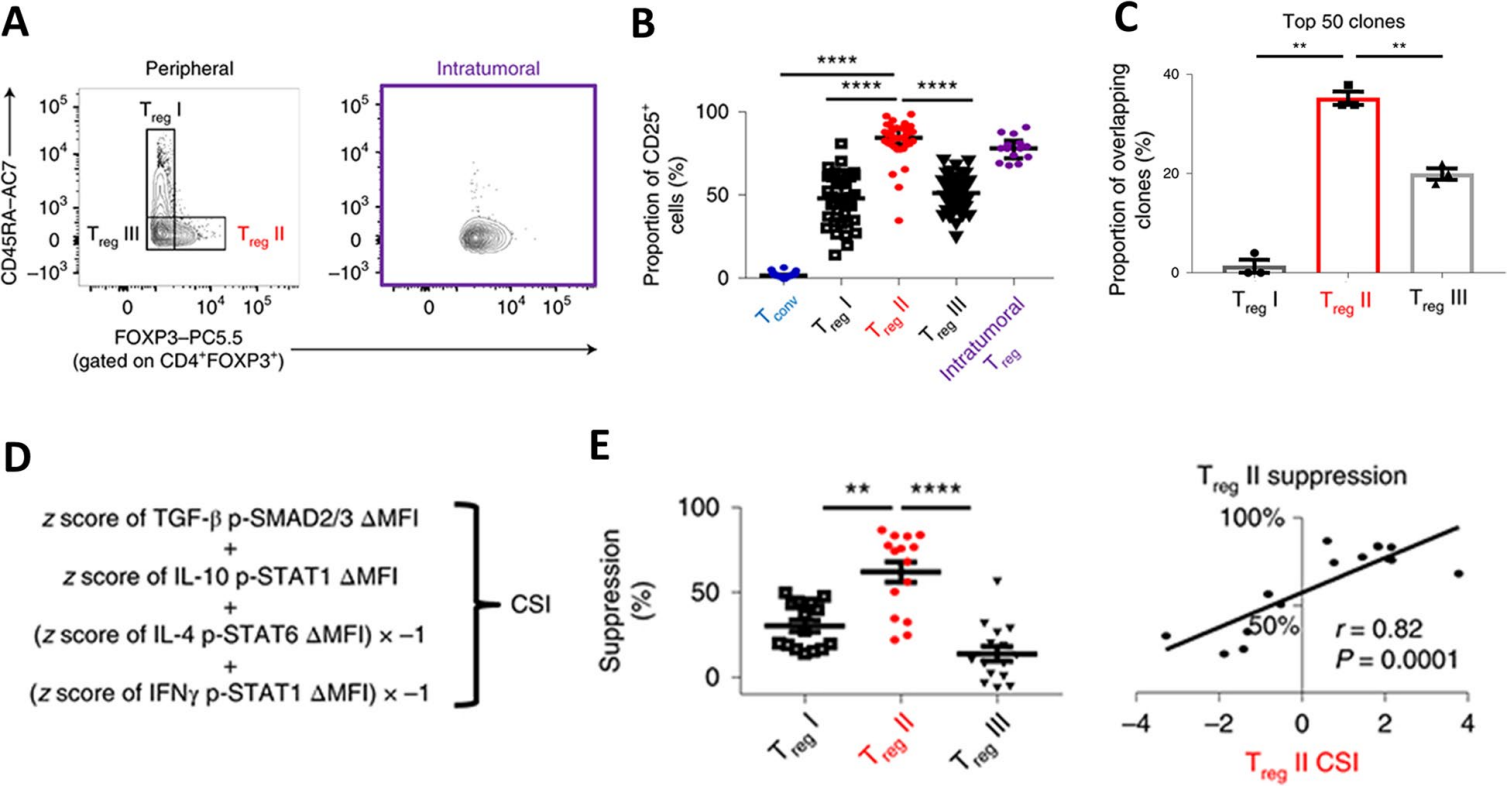
Association of signaling responsiveness in regulatory T cell (Treg) II cells with intratumoral Tregs and suppressive function. Peripheral Treg populations were defined based on differential expression of CD45RA and FoxP3 and compared to intratumoral Tregs **A**. Peripheral Treg populations were compared to intratumoral Tregs for expression of CD25 and other markers (not shown) **B**. T cell receptor sequencing was performed and the proportion of overlapping clones among the top 50 clones was compared between peripheral Treg populations and matched intratumoral Tregs **C**. A cytokine signaling index (CSI) was calculated from the z-score of the difference in median fluorescent intensities of cytokine-stimulated minus unstimulated groups **D**. Suppression of responder T cells (CD4^+^CD45RA^+^CD25^−^) by Treg I, II, and III cells was compared, and Treg II cell suppression was correlated with the Treg II CSI **E**. Modified from Wang et al., Nat Immunol. 2019 [47]. Copyright© 2019, Springer Nature

As few studies have been published on the association between signaling responsiveness in TILs and PBMCs, limited data exist on similarities between the two compartments. However, similarities have been observed in the signaling responsiveness and function of a specific population of peripheral blood Tregs and intratumoral Tregs in breast cancer patients [47]. In this study, peripheral Tregs were categorized into three distinct subgroups: Treg I (CD45RA^hi^ FoxP3^lo^), II (CD45RA^lo^ FoxP3^hi^), and III (CD45RA^lo^ FoxP3^lo^) (Fig. 3A). Treg II cells in the periphery of breast cancer patients were found to be most phenotypically like intratumoral Tregs via expression of CD25 and other markers (Fig. 3B) and T cell receptor clonal overlap (Fig. 3C). Signaling responsiveness was tested by phosphoflow cytometry in conventional T cells, total Tregs, and Treg I, II, and III cells; Treg II cells were found to be more sensitive to immunosuppressive cytokines (IL-10-induced p-STAT1 and TGF-β-induced p-SMAD2/3) and less responsive to immunostimulatory cytokines (IL-4-induced p-STAT6 and IFN-γ-induced p-STAT1) compared to the other cell types evaluated. The combined effect of cytokine signaling response was calculated with a cytokine signaling index (CSI), in which a higher CSI indicated more responsiveness to immunosuppressive cytokines and less responsiveness to immunostimulatory cytokines (Fig. 3D). There was no association between plasma levels of IL-10, TGF-β, IL-4, and IFN-γ and signaling responsiveness. Association between signaling responsiveness and function were investigated; Treg II cells were more suppressive of responder T cells (CD4^+^CD45RA^+^CD25^−^) than Treg I and Treg III cells, and Treg II cells with a higher CSI correlated with increased suppressive ability (Fig. 3E).

Connections between impaired IFN-γ signaling responsiveness via p-STAT1 in peripheral monocytes and tumor associated macrophage (TAM) infiltration in paired primary tumors have been noted in patients with non-metastatic breast cancer [48]. Patients with lower IFN-γ signaling responsiveness in peripheral monocytes had lower levels of TAM infiltration. TAMs are known to be recruited to tumors through the expression of CSF1R. A negative correlation was found between the expression of CSF1R on monocytes and the responsiveness of peripheral monocytes to IFN-γ signaling, which provides a potential mechanism for the reduced infiltration of TAMs in the tumors of these patients.

In summary, while limited studies have explored the association between signaling responsiveness in TILs and PBMCs, similarities have been observed in the signaling responsiveness and function of a specific population of peripheral blood Tregs and intratumoral Tregs in breast cancer patients. Furthermore, impaired IFN-γ signaling in peripheral monocytes may contribute to reduced TAM infiltration in non-metastatic breast cancer patients, potentially mediated by the expression of CSF1R on monocytes.

### Phosphoflow cytometry to determine the effect of agents on the immune system

#### High-dose IL-2

Phosphoflow cytometry has been used as a tool to determine the effect of cancer treatment agents on the immune system. High-dose IL-2 treatment is employed to cancer patients with the goal of activating immune cells to destroy cancer cells. The activation of signaling pathways in immune cells in response to IL-2 therapy has been studied using phosphoflow cytometry. In one study, PBMCs from 11 patients with metastatic melanoma and renal cell carcinoma were analyzed for p-STAT5 at baseline and 1 h after high-dose IL-2 [49]. The activation of p-STAT5 in immune cells varied between individuals but persisted in CD4^+^ and CD8^+^ T cells and CD56^+^ NK cells up to 3 weeks in patients who responded clinically to treatment. In another study, 17 patients with metastatic renal cell carcinoma and melanoma were treated with high-dose IL-2 and sorafenib, a kinase inhibitor, and activation of p-STAT5 in peripheral T cells was assessed [50]. Elevated p-STAT5 was noted in CD4^+^ and CD8^+^ T cells 1 h after IL-2 administration and was not altered by the addition of sorafenib. The findings mentioned here highlight the significance of using phosphoflow cytometry to understand the effect and efficacy of a cancer immunotherapy agent on the immune system.

#### STAT3 inhibition

Targeting immune cell signaling has been explored as a way to enhance clinical response to immunotherapy. One study examined the ability of the STAT3 inhibitor WP1066 to enhance T cell anti-tumor activity through Treg inhibition in patients with melanoma brain metastases [51]. The baseline percentage of p-STAT3^+^ PBMCs was higher in melanoma patients compared to healthy donors. WP1066 reduced IL-6-induced p-STAT3 expression and enhanced CD3^+^ T cell cytotoxicity against melanoma. The response was dependent on the presence of Tregs, as WP1066 was able to inhibit FoxP3^+^ Treg induction through the inhibition of p-STAT3, contributing to the anti-tumor response. These findings suggest that specifically targeting relevant immune cell signaling pathways may be a promising approach for enhancing the effectiveness of cancer treatment.

### Signaling in peripheral blood immune cells to predict cancer patients’ response to treatment and outcome

Signaling pathways activated in PBMCs have been studied as a way to predict cancer patient response to treatment and outcome. Studies reporting associations of phosphoflow cytometry-detected cytokine signaling pathways and cancer patient response are summarized in Table 2.

**Table 2:** Cytokine signaling pathways associated with survival outcome following treatment in cancer patients

Cancer type	Cell type	Baseline or change after therapy	Treatment	Outcome measurement	Cytokine and phosphorylated signaling protein	Association with outcome	Reference
Melanoma	Lymphocytes	Change after therapy	High-dose interferon therapy	Disease-free survival, overall survival	IFN-α, p-STAT1(Y701)	+	[52]
Stage III/IV Melanoma	Regulatory T cells, CD8^+^ T cells	Change after therapy	Nivolumab	Overall survival	p-STAT3(S727)	+	[53]
Breast	CD4^+^ naïve T cells	Baseline	Standard of care	Relapse-free survival	IL-6, p-STAT1(Y701)	+	[19]
					IL-6, p-STAT3(Y705)	+	
	Monocytes	Baseline	Standard of care	Relapse-free survival	IFN-γ, p-STAT1(Y701)	+	[48]
	CD45RA^lo^FoxP3^hi^ Regulatory T cells (Treg II)	Baseline	Standard of care	Relapse-free survival	TGF-β, p-SMAD2(S465/S467) p-SMAD3(S423/425)	-	[47]
					IL-10, p-STAT1(Y701)	-	
					IL-4, p-STAT6(Y641)	+	
					IFN-γ, p-STAT1(Y701)	+	

#### Association of phosphoflow cytometry with clinical outcome in melanoma

**Fig. 4:**
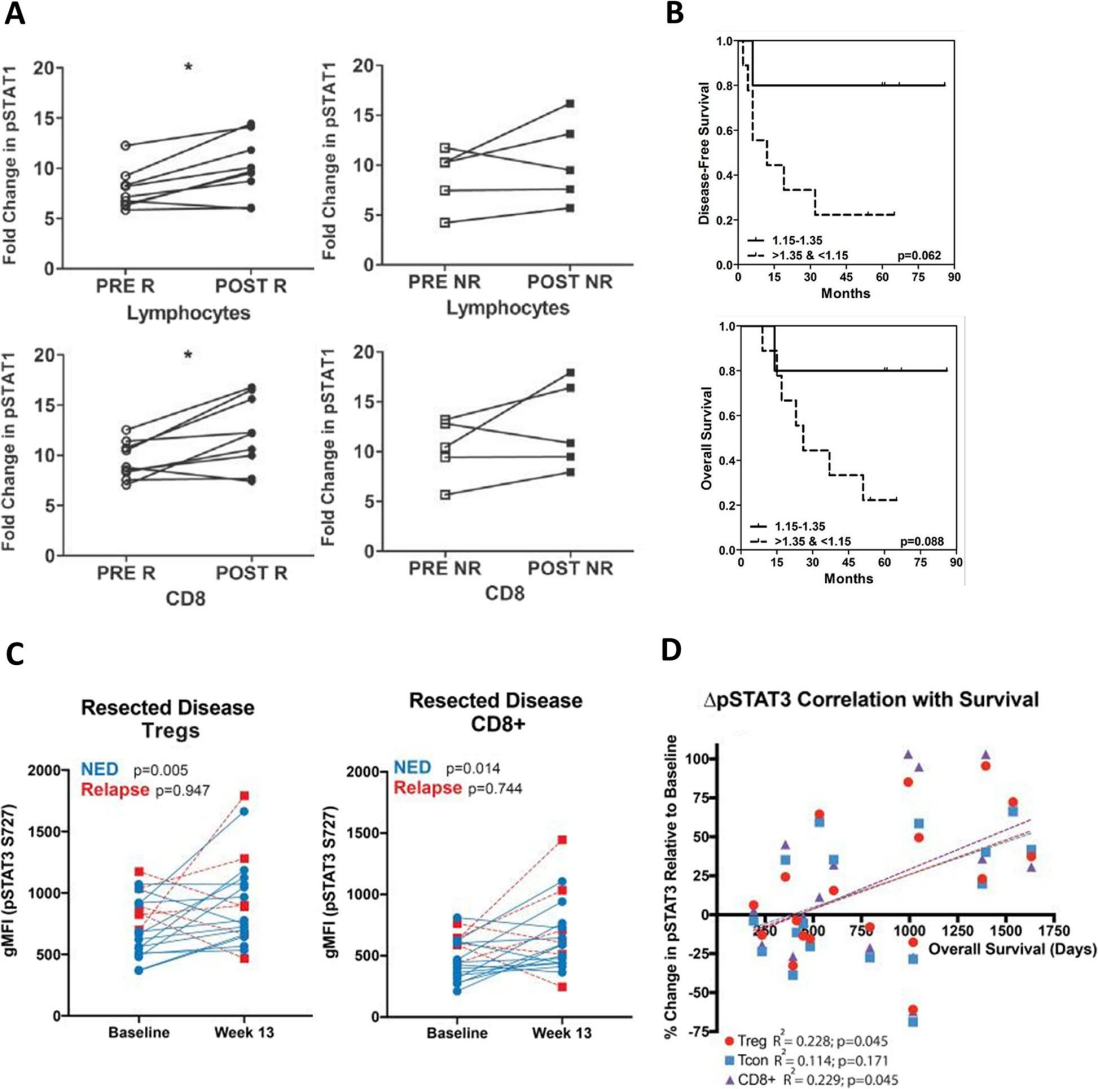
Associations of phosphoflow cytometry signaling with outcome in melanoma patients. In panels A and B, 14 melanoma patients were treated with high-dose IFN-α (HDI) and p-STAT1 signaling responsiveness to IFN-α was analyzed both prior to and after treatment. p-STAT1 induction at baseline and after 4 weeks of treatment was analyzed in total lymphocytes and CD8^+^ T cells from responding (R) and non-responding (NR) patients **A**. The relationship between IFN-α-induced p-STAT1 activation and clinical outcome was assessed by Kaplan–Meier analysis by generating a ratio (post/pre) of p-STAT1 fold induction in lymphocytes before and after treatment, and comparing patients above and below the median **B**. In a separate study, in panels C and D, melanoma patients were analyzed for p-STAT3 expression after surgical resection and adjuvant nivolumab. The geometric mean fluorescent intensity (gMFI) of p-STAT3 from baseline to week 13 in regulatory T cells (Tregs) and CD8^+^ T cells was compared between patients with no evidence of disease (NED) and relapse **C**. The correlation between overall survival and percent change in p-STAT3 at week 13 compared to baseline was assessed in Tregs, conventional T cells (Tcon), and CD8^+^ T cells **D**. Panels A and B are modified from Simons et al., J. Transl Med. 2011 [52]. Copyright© 2011, Simons et al., Licensee BioMed Central Ltd. Panels C and D are modified from Woods et al., Clin Cancer Res. 2018 [53]. Copyright© 2018, American Association for Cancer Research

The association of signaling responsiveness using phosphoflow cytometry and cancer patient outcome has been studied in patients with melanoma. Phosphoflow cytometry was used to analyze p-STAT1 expression in PBMCs from 17 healthy donors and 19 melanoma patients before and after treatment with IFN-α [16]. Healthy donors had higher basal levels of p-STAT1 in total PBMCs, NK cells, and T cells compared to melanoma patients. P-STAT1 was detected in PBMCs of two patients receiving IFN-α treatment and increased with treatment in one patient. In a later study, 14 melanoma patients were treated with high-dose IFN and assessed for signaling responsiveness through phosphoflow cytometry of IFN-α-induced p-STAT1 in peripheral blood lymphocytes [52]. Patients who responded clinically to treatment had a significant increase in IFN-α signaling capacity from days 0 to 29 in total lymphocytes and CD8^+^ T cells, which was not seen with non-responders (Fig. 4A). In addition, p-STAT1 response to IFN-α was compared to disease-free and overall survival. Patients with a greater increase in signaling responsiveness to IFN-α post-treatment had a trend towards better outcome (Fig. 4B). These findings suggest that defects in IFN signaling can be overcome with high-dose IFN therapy in some patients, and that early changes in cytokine signaling detected by phosphoflow cytometry may predict response to therapy.

In another study, PBMC samples from melanoma patients were analyzed for p-STAT3 expression after surgical resection and adjuvant nivolumab treatment [53]. An increase in p-STAT3^+^ Tregs and CD8^+^ T cells at 13 weeks compared to baseline was observed in non-relapsing patients, but not in relapsing patients (Fig. 4C). In addition, conventional T cells from non-relapsing patients had upregulation of p-STAT3. Similar results were seen in unresectable stage III/IV melanoma patients treated with nivolumab. The change in p-STAT3 at week 13 compared to baseline in both Tregs and CD8^+^ T cells from patients with metastatic disease was also positively correlated with survival (Fig. 4D). In vitro experiments revealed that PD-1 blockade increased p-STAT3 expression in Tregs, conventional T cells, and CD8^+^ T cells. This induction of p-STAT3 was accompanied by a reduction in Treg suppressive capacity.

Overall, phosphoflow cytometry findings in melanoma patients indicate that signaling, as measured by p-STAT1 and p-STAT3 expression, can potentially be used to predict patient response to high-dose IFN-α and nivolumab therapies, respectively, with increased signaling correlating with better treatment response.

#### Association of phosphoflow cytometry with outcome in breast cancer

**Fig. 5:**
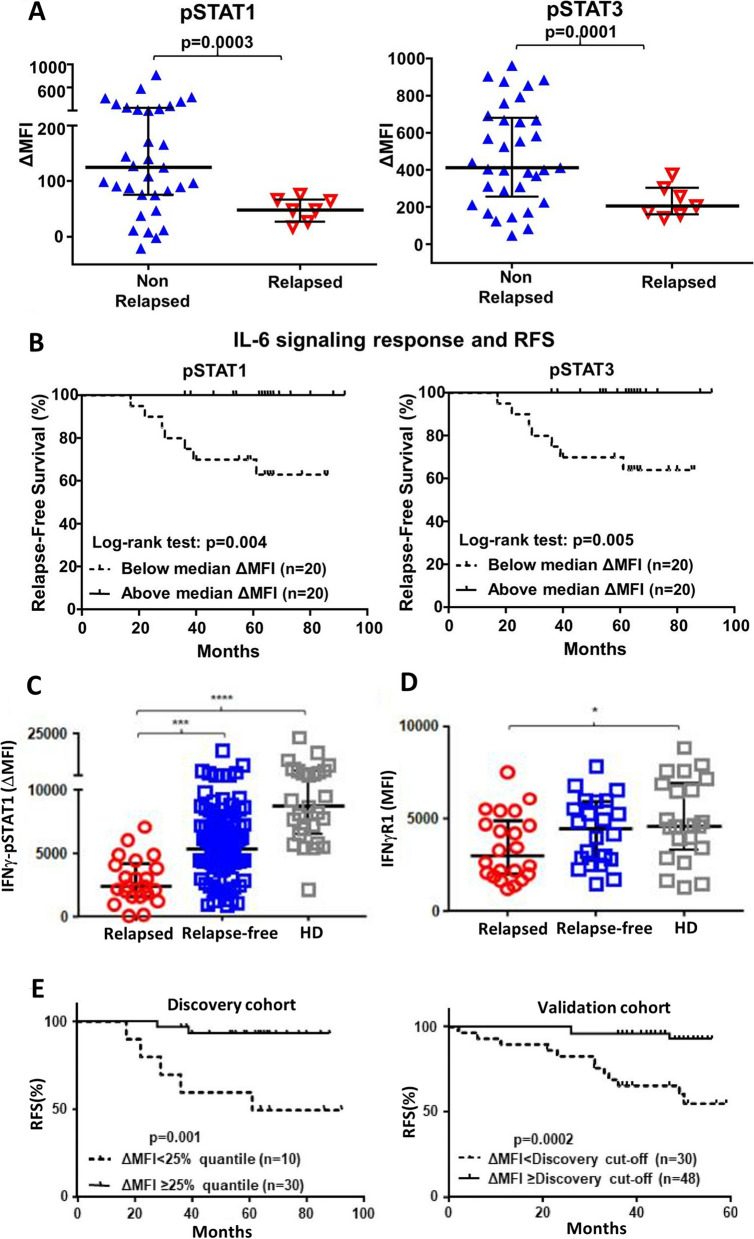
Associations of phosphoflow cytometry signaling with outcome in breast cancer patients. In panels A-B, IL-6 signaling response was assessed in peripheral blood CD4^+^ naïve T cells prior to any therapy. The delta median fluorescent intensity (ΔMFI) in p-STAT1 and p-STAT3 response to IL-6 was assessed between non-relapsed and relapsed patients **A**. The signaling responsiveness (ΔMFI) above and below the median was also compared by Kaplan–Meier analysis to assess relapse-free survival **B**. In panels C-E, IFN-γ signaling response was assessed in peripheral monocytes from breast cancer patients prior to therapy. IFN-γ p-STAT1 signaling responsiveness in peripheral blood monocytes of breast cancer patients associates with relapse and relapse-free survival. MFI of pSTAT1 in monocytes stimulated with IFN-γ minus unstimulated in relapsed and relapsed-free breast cancer patients compared to healthy donors (HD) **C**. MFI of IFNγR1 on monocytes in relapsed and relapsed-free breast cancer patients compared to healthy donors **D**. Kaplan–Meier survival curves of relapse-free survival (RFS) between patients with high (≥ 25% quantile) vs low (< 25% quantile) IFN-γ responsiveness in both a discovery and validation cohort **E**. Panels A and B are modified from Wang et al., Cancer Res. 2017 [19]. Copyright© 2017. American Association for Cancer Research. Panels C-E are modified from Wang et al., EBioMedicine 2020 [48]. © Wang et al. Published by Elsevier B.V

Other studies have identified a relationship between cytokine signaling pathways in PBMCs as measured by phosphoflow cytometry and clinical outcome in breast cancer. Wang et al. found that impaired IL-6 p-STAT1 and p-STAT3 activation in peripheral T cells was observed at baseline in breast cancer patients who later relapsed compared to patients who did not relapse following standard treatment (Fig. 5A) [19]. In addition, higher levels of IL-6-induced p-STAT1 and p-STAT3 (above the median) significantly associated with improved relapse-free survival (Fig. 5B). Another study investigated IFN-γ signaling response via p-STAT1 in peripheral monocytes from breast cancer patients who later relapsed or remained relapse-free, and compared them to healthy donors [48]. In that study, IFN-γ-induced p-STAT1 in peripheral monocytes was lower in breast cancer patients who relapsed compared to those who remained relapse-free, as well as compared to healthy donors (Fig. 5C). Additionally, IFN-γ Receptor1 levels were higher in healthy donor monocytes compared to relapsed breast cancer patients (Fig. 5D). Lower IFN-γ signaling response in peripheral blood monocytes at diagnosis correlated with worse relapse-free survival (RFS), and was independent of clinicopathologic features and plasma IFN-γ levels (Fig. 5E). The researchers also found a significant positive correlation between IFN-γ-p-STAT1 in monocytes and IL-6-p-STAT1/3 in CD4^+^ naïve T cells.

In another study in breast cancer patients, signaling responsiveness was investigated in peripheral Treg II cells, which were identified as being most phenotypically similar to intratumoral Tregs [47]. Breast cancer patients with increased signaling responsiveness to immunosuppressive cytokines (IL-10 and TGF-β) in Treg II cells had worse RFS, whereas patients with increased signaling responsiveness to immunostimulatory cytokines (IL-4 and IFN-γ) in Treg II cells had better RFS. Calculation of a cytokine signaling index, by combining signaling responsiveness to all four cytokines, determined that patients with peripheral Treg II cells with a higher cytokine signaling index, indicative of increased sensitivity to immunosuppressive cytokines and decreased sensitivity to immunostimulatory cytokines, had a worse RFS.

To summarize, phosphoflow cytometry studies in breast cancer patients have revealed that impaired activation of cytokine signaling pathways, such as IL-6-induced p-STAT1 and p-STAT3 in peripheral T cells, and lower IFN-γ-induced p-STAT1 in peripheral monocytes, are associated with increased risk of relapse. In addition, increased signaling responsiveness in Treg II cells to immunosuppressive cytokines and decreased signaling responsiveness to immunostimulatory cytokines is linked to worse relapse-free survival. Further investigation of the association of phosphoflow cytometry signaling in peripheral immune cells with cancer patient outcome is warranted.

### Phosphoflow cytometry in preclinical mouse models

There have been several studies that have used phosphoflow cytometry to investigate signaling pathways in peripheral blood of mice, albeit fewer than in humans. These include investigation of T cell receptor signaling, phosphorylated signaling proteins in B cell subpopulations, the activation of signaling pathways in peripheral blood cells of mice with various diseases, and a tumor-targeting vaccine. Phosphoflow cytometry was used to analyze the differences in T cell receptor signaling between Tregs and CD4^+^ T cells [9]. The study found that Treg and non-Treg CD4^+^ T cells displayed differences in TCR-dependent signaling responses following in vitro or in vivo stimulation. The researchers used phosphoflow cytometry to profile the kinetics and extent of TCR signaling (ZAP-70 and PKC-0 phosphorylation) in Tregs and non-Tregs. The experiments were performed using cells from 6–8‒week-old C57BL/6J mice or OT-II TCR transgenic mice, with cells harvested from both lymph nodes and spleens. Another study developed a phosphoflow protocol to evaluate the status of phosphorylated signaling proteins in murine and human B cell subpopulations [54].

Phosphoflow cytometry has also been employed in mouse models of various disease states, including graft-versus-host disease [55], aortic valve stenosis [56], leukemia [57] and poxviral infection [58]. In a mouse model of aortic valve stenosis, researchers assayed SMAD2/3 phosphorylation in circulating leukocytes and platelet-leukocyte aggregates and found that p-SMAD2/3 staining was more intense in leukocytes of hypercholesterolemic mice that developed aortic valve stenosis, suggesting increased circulating active TGF-β1 levels [56]. In the study on poxviral infection, researchers found that STAT1 and STAT3 pathways were rapidly activated in C57BL/6 mice resistant to poxviral infection, whereas in susceptive BALB/c mice, IL-6-dependent STAT3 activation did not occur [58]. Phosphoflow cytometry has been investigated in mouse models of blood-based cancers [59, 60], but it has not yet been studied in mouse models of solid tumors. It has, however, been applied to the investigation of the activation of MUC-1 specific T cells following vaccination with a MUC-1 targeted vaccine in non‒tumor bearing mice [61]. The investigators found that MUC1-specific T cells from MUC1-transgenic mice 3 h after a dendritic cell vaccination showed a higher level of p-ZAP-70, p-ERK1/2, and p-PKC-theta phosphorylation compared to non-transgenic mice. MUC-1 is the target of various cancer vaccines currently in development [62].

Despite the small number of studies, phosphoflow cytometry has shown promise in the examination of immune responses and signaling pathways in mouse models. This includes the evaluation of the effects of anti-cancer therapeutics on signaling in immune cells and the investigation of defective immune cell signaling in various disease states. However, since there have been no studies utilizing phosphoflow cytometry in preclinical solid tumor mouse models, there is need for further research in this area. The use of this method has the potential to improve our understanding of cancer biology and evaluate the effectiveness and mechanism of action of potential treatments, including immunotherapies.

## Conclusions

Phosphoflow cytometry is a useful technique for studying the activation of signaling pathways and the sensitivity of these pathways to cytokine stimulation in peripheral immune cells. Herein, to our knowledge, is the first review of the application of phosphoflow cytometry in the field of cancer immunology. The use of phosphoflow cytometry in peripheral blood immune cells allows for dynamic and non-invasive monitoring of the activation of signaling pathways in immune cells at baseline and changes over the course of disease and treatment, which may not be as easily accessible with other methods. Many of the limitations that existed when phosphoflow cytometry was initially developed, restricting its potential use as a widespread immunomonitoring tool, have since been addressed. Further studies, including Good Laboratory Practice (GLP) applications, will be required to apply phosphoflow cytometry as a method in clinical practice. In addition, more work is needed to optimize the type of cytokine stimulation and flow cytometry panels needed to measure the key signaling pathways that are relevant to the physiologic state/indication being evaluated. Cytokines and the signaling pathways they activate are essential for regulation of the immune response. Therefore, this method goes beyond providing basic information on immune cell numbers and/or phenotype and may inform on the function of immune cells by identifying activated and defective signaling pathways.

Despite the potential value of phosphoflow cytometry for understanding immune responses in cancer patients, its use in this context has been relatively limited. In this review, we show that phosphoflow cytometry has been applied to study a number of important cytokine signaling pathways in peripheral blood of cancer patients, including signaling induced by interferon, IL-6, and IL-7. However, there are additional key signaling pathways in cancer immunology that have not been explored with phosphoflow cytometry, including signaling related to immune checkpoints such as PD-1, PD-L1, and CTLA-4, NF-kB, Wnt/β-catenin, PI3K-Akt-mTOR, STING, the adenosine pathway, and additional cytokines activating the JAK/STAT signaling cascade such as IL-12 and IL-15 [63–69]. Application of phosphoflow cytometry to these additional pathways represents an opportunity for further research, as the mechanisms underlying response to immunotherapy in cancer patients are often poorly understood. By investigating numerous signaling pathways in peripheral immune cells, it may be possible to identify therapeutic targets that could restore normal immune cell function and improve treatment outcomes, and help in the dynamic assessment of patient response to therapy prior to and during the therapeutic regimen.

## Data Availability

Not applicable.

## Abbreviations

IHC: Immunohistochemistry
IF: Immunofluorescence
IP: Immunoprecipitation
RT-qPCR: Real-time quantitative PCR
PBMC: Peripheral blood mononuclear cell
Treg: Regulatory T cell
IFN-γ: Interferon gamma
NK: Natural killer
Th1: T helper 1
IFN-α: Interferon alpha
ISG: Interferon-stimulated genes
JAK: Janus kinase
STAT: Signal transducer and activator of transcription
IL-6: Interleukin 6
IL-6R: Interleukin 6 receptor
Th2: T helper 2
NSCLC: Non-small cell lung cancer
HCC: Hepatocellular carcinoma
IL-7: Interleukin 7
IL-7R: Interleukin 7 receptor
IL-7Rα: Interleukin 7 receptor alpha
TCR: T cell receptor
TILs: Tumor infiltrating lymphocytes
CRC: Colorectal cancer
CSI: Cytokine signaling index
TAM: Tumor associated macrophage
RFS: Relapse-free survival
